# Preliminary data from a survey about nutrition in mental health

**DOI:** 10.1192/j.eurpsy.2025.1606

**Published:** 2025-08-26

**Authors:** A. Litta, A. Nannavecchia, V. Favia, M. V. Minò, A. Vacca

**Affiliations:** 1 Department of Precision and Regenerative Medicine and Ionian Area (DiMePRe-J) University of Bari “Aldo Moro”; 2 AReSS Puglia- Regional Strategic Agency for Health and Social; 3 Istituto Tumori Giovanni Paolo II, IRCCS, National Cancer Institute, Bari; 4 Psychiatric Rehabilitation Center “Don Tonino Bello”- Assoc. M.I.T.A.G. Onlus, Brindisi; 5 Mental Health Department, ASL Taranto, Taranto, Italy

## Abstract

**Introduction:**

Diet-related support is urgently needed for people with serious mental illness underlying the role of nutrition in a biopsychosocial approach. Integrating healthy lifestyle practices such as a balanced diet and physical exercise could supplement and amplify the effects of existing pharmacotherapies and psychotherapies. Despite its considerable role, nutritional literacy of mental health professionals appears scarce. Specific nutrition training courses for mental health professionals are needed in order to increase awareness on nutrition as a well-being contributing factor in the biopsychosocial model.

**Objectives:**

The aim of this study is to investigate insight and attitudes on nutrition in mental health among a sample of psychiatrists and psychologists resident in Italy.

**Methods:**

The survey was conducted from May to June 2024 and the questionnaire was anonymous and self-rated, accessible via Google forms. The sample comprised 110 Italian mental health professionals (adult and child psychiatrists, psychologists) who voluntarily completed the on‑line questionnaire.

**Results:**

110 participants (61 psychologists, 46 adult psychiatrists and 3 child psychiatrists) agreed to participate to our survey. 89.2 % (n= 91) of them worked in southern Italy.The majority of participants were female (77.98%) and reported working in a Mental Health Centre (n= 41, 37,27%), followed by psychiatric residential facilities (n=22, 20%), private practice (n= 19, 17.27%), hospital (n= 14, 12.73%), university research centre (n=4. 3.64%), other non specified institute (n=10; 9.09%). The numer of years of working as mental health professional was 19.27 ± 11.27 years. Adopting “sometimes” nutritional approach for the treatment of the patients was the answer mostly reported (n= 56, 51.4%) followed by “most of the time” (n= 28, 25.69%), “always” (n=11; 10.09%), “never” (n= 7, 6.42%), “almost never “(n= 7, 6.42%).

**Conclusions:**

Despite the scientific evidence and some treatment guidelines in support of this relationship, the implementation of nutritional psychiatry into routine clinical practice remains limited. Integrating nutrition into the clinical practice of psychologists and psychiatrists and providing evidence-based nutritional advice represent an interesting mental health challenge to address in the coming years.

**Disclosure of Interest:**

None Declared

